# Associations between appetite loss and clinical features as well as inflammatory cytokines in adolescents with major depressive disorder

**DOI:** 10.3389/fpsyt.2025.1583060

**Published:** 2025-05-14

**Authors:** Lewei Liu, Xi Zhang, Jinyue Xue, Lili Zhao, Pei Tang, Yinghan Tian, Haojie Fan, Mingru Hao, Xin Zhao, Feng Geng, Daming Mo, Lei Xia, Huanzhong Liu

**Affiliations:** ^1^ Department of Psychiatry, Chaohu Hospital of Anhui Medical University, Hefei, Anhui, China; ^2^ Department of Psychiatry, Affiliated Psychological Hospital of Anhui Medical University, Hefei, Anhui, China; ^3^ Department of Psychiatry, School of Mental Health and Psychological Sciences, Anhui Medical University, Hefei, Anhui, China; ^4^ Department of Psychology and Sleep Medicine, The Second Affiliated Hospital of Anhui Medical University, Hefei, Anhui, China

**Keywords:** appetite loss, clinical features, inflammatory cytokines, adolescents, major depressive disorder

## Abstract

**Background:**

Appetite loss is common in major depressive disorder (MDD). However, the psychosocial and biological mechanisms behind appetite loss remain unclear, particularly in the adolescent MDD population. Therefore, this study aimed to examine the links between appetite loss and clinical symptoms as well as inflammatory cytokines levels in this population.

**Methods:**

Between January and December 2021, this study included 171 depressed adolescents. A range of scales were used to assess the patients’ clinical symptoms, including depression severity, negative life events, insomnia, and alexithymia. Additionally, plasma inflammatory cytokines levels were measured, including interleukin (IL)-1β, IL-6, IL-10, IL-17A and tumor necrosis factor-α (TNF-α).

**Results:**

The prevalence of appetite loss among adolescents with MDD was as high as 76.0%. Univariate analyses showed that patients with appetite loss had higher scores of the Hamilton Depression Scale (HAMD), interpersonal relationships, study pressure, punishment, sense of loss, the Insomnia Severity Index Scale (ISI) and difficulty identifying feelings, as well as higher levels of Log IL-6 (all *p* < 0.05) Furthermore, regression analyses revealed that appetite loss was independently associated with HAMD score (OR = 1.158, 95% CI = 1.091-1.229, *p* < 0.001), punishment score (OR = 1.117, 95% CI = 1.039-1.201, *p* = 0.003), and Log IL-6 level (OR = 5.041, 95% CI = 1.137-22.344, *p* = 0.033).

**Conclusion:**

Adolescents with MDD face an elevated risk of appetite loss, which may correlate with clinical symptoms such as depression severity and negative life events, as well as elevated IL-6 level. Healthcare professionals should target these risk factors, including inflammation, to mitigate appetite loss.

## 1 Introduction

Major depressive disorder (MDD) is a severe mental disorder projected to become the leading cause of disability globally by 2030 (1). In recent years, driven by social changes and increased academic stress, MDD has emerged as the most common affective disorder among adolescents (2). According to the latest epidemiological survey, the prevalence of MDD among Chinese children and adolescents has been as high as 2.0% (3). Compared to adults, MDD in adolescents is characterized by a longer disease course, more severe symptoms, and higher rates of recurrence and disability (4). In addition, changes in appetite are one of the core symptoms of MDD, with the direction varying according to the subtype. Typically, MDD is associated with appetite loss and weight loss (5). A recent cross-sectional study found that 56.5% of adults with MDD exhibited appetite loss (6). While adolescence is a critical period for the development of eating disorders, adolescents with MDD are more susceptible to a range of maladaptive eating behaviors due to their immature psychocognitive development (7). However, there is a notable lack of studies specifically exploring appetite loss in adolescents with MDD. Co-occurring appetite loss not only poses significant challenges to the treatment of MDD, but also seriously affects patients’ prognosis, growth and development. For example, a secondary analysis of randomized trials found that MDD patients with appetite loss had a poorer treatment response (8). Kitagawa and colleagues also identified appetite loss as a predictor of suicidal ideation and self-harm in adolescents (9). Therefore, understanding the mechanisms underlying appetite loss in adolescents with MDD is crucial for early screening, prevention and optimal treatment in this population.

Previous studies have shown that appetite loss in patients with MDD is associated with sociodemographic, clinical, and biochemical factors. Specifically, it may be linked to sociodemographic factors such as gender, age, and educational level. Additionally, appetite loss is also related to patients’ mental health symptoms. In adolescents with MDD, depressed mood can directly influence eating behaviors. There is evidence that a depressed mood can influence food choices by affecting appetite or altering food availability (10). A recent case-control study found that patients with decreased appetite symptoms also exhibited more severe depressive symptoms (11). Furthermore, research has indicated that stress resulting from negative life events can significantly affect eating behaviors of patients with MDD. For example, a longitudinal study showed that appetite loss was significantly associated with adverse life events (particularly the death of family members and loss of romantic relationships) in depressed adults (12). Also, studies on patients with eating disorders have revealed that both alexithymia and depressive symptoms are more pronounced in individuals with anorexia nervosa than in those with bulimia nervosa (13). Another study found that adolescent anorexia nervosa was also associated with insomnia symptoms (14). However, most of the studies mentioned above have focused on adults with MDD or on eating disorder groups. Therefore, there is a need to explore the psychological factors associated with appetite loss in adolescents with MDD, providing a theoretical basis for early clinical intervention.

Importantly, the combination of MDD with appetite loss may involve complex biological mechanisms. One of the hot topics in recent years is the role of inflammatory cytokines. A study of hospitalized elderly patients showed that appetite loss was significantly associated with elevated levels of several serum inflammatory cytokines, including interleukin (IL)-1β, IL-6, and IL-33 (15). Guo and colleagues found elevated levels of inflammatory cytokines in MDD patients with decreased appetite and suggested that this elevated inflammation might be caused by an abnormal gut microbiota (6). However, there were also studies that came to inconsistent conclusions. Okamoto and colleagues conducted a small-sample cross-sectional study (n = 40) and found that decreased appetite was associated with lower high-sensitivity C-reactive protein (hsCRP) levels in patients with MDD (16). Such inconsistencies may be attributed to the heterogeneity of sample sizes, study populations, and assessment tools. Therefore, additional relevant studies are needed.

This study aimed to preliminarily explore the associations between appetite loss and clinical symptoms (including depression severity, negative life events, insomnia, and alexithymia) as well as inflammatory cytokines levels in adolescents with MDD. And the goal was to provide a theoretical basis for understanding the mechanisms of appetite loss in this population and to inform early clinical interventions.

## 2 Methods

### 2.1 Study design and participants

This research was conducted as a cross-sectional study. Between January and December 2021, we enrolled adolescents diagnosed with MDD at two medical centers in Hefei, Anhui Province, China: the Chaohu Hospital of Anhui Medical University and the Fourth People’s Hospital of Hefei. Participants were required to meet the following criteria: (1) aged between 12 and 18 years; (2) diagnosed with MDD based on the Diagnostic and Statistical Manual of Mental Disorders, fifth edition (DSM-5); and (3) willing to actively engage and collaborate throughout the study period. Individuals were excluded if they met any of the following conditions: (1) diagnosed with other mental disorders such as schizophrenia or bipolar disorder according to DSM-5; (2) had major infections, autoimmune diseases, or other significant health issues that could influence the study outcomes; and (3) were currently undergoing treatment with anti-inflammatory drugs.

This study commenced after receiving ethical review and approval from the Ethics Committee of the Chaohu Hospital of Anhui Medical University, under reference number 202009-KYXM-04. All procedures were conducted in accordance with the ethical standards of the institutional and national research committee and with the 1964 Helsinki Declaration and its later amendments or comparable ethical standards. Before enrolling participants, we provided a detailed explanation of the study procedures to both the participants and their parents or legal guardians, and obtained their written informed consent.

### 2.2 Measuring Instruments

#### 2.2.1 Sociodemographic characteristics

In this study, participants were asked to provide various sociodemographic details through a custom-designed survey. The information gathered included their gender, age, body mass index (BMI), age at onset, duration of illness, and types of antidepressants.

#### 2.2.2 Depressive symptoms

**Figure 1:**
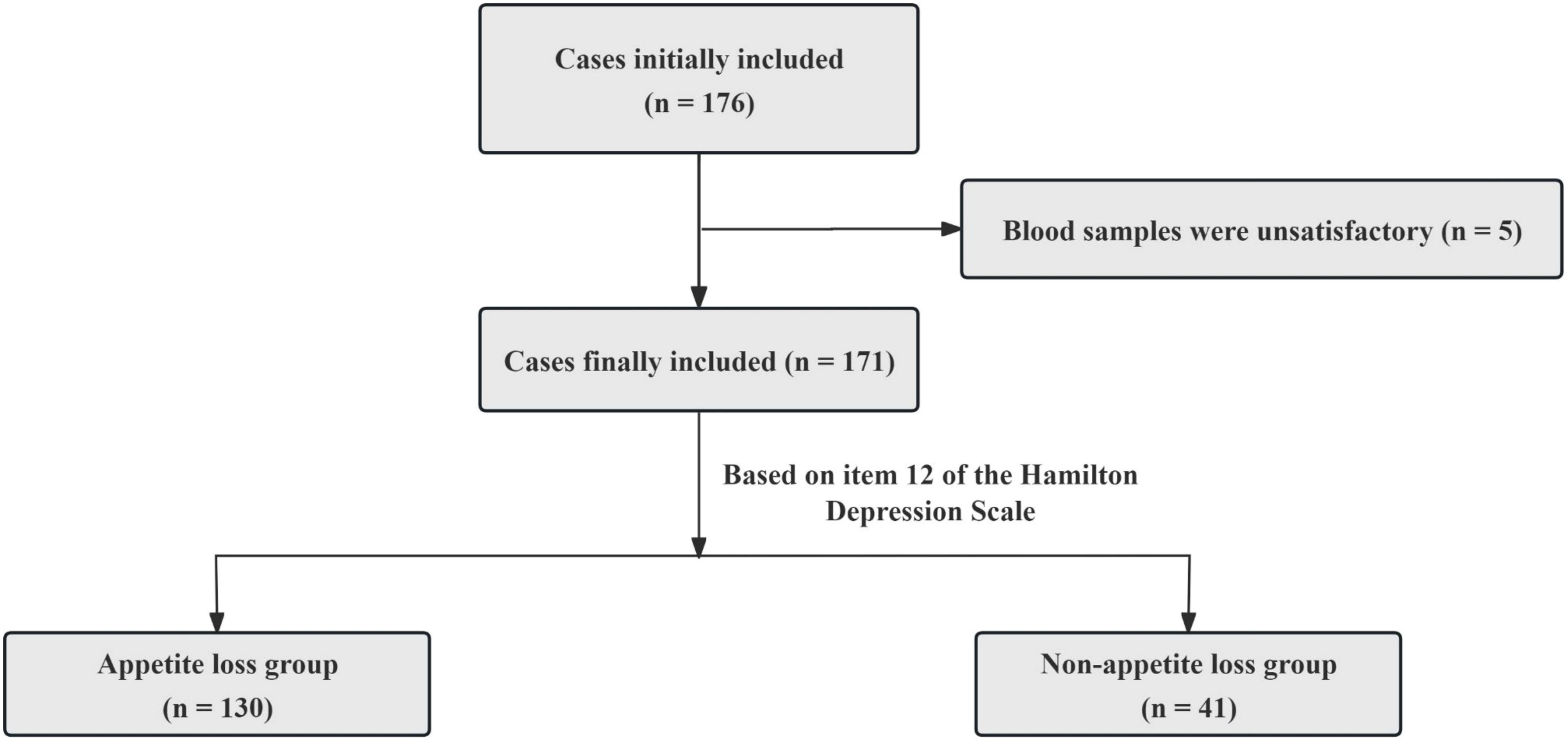
Case screening flowchart.

The Hamilton Depression Scale (HAMD) was employed to evaluate the severity of depressive symptoms exhibited by the patients over the past week (17). The HAMD consists of 24 items, with scores ranging from 0 to 76. An elevated total score indicates a greater severity of depressive symptoms. Additionally, patients were categorized into two groups based on item 12 of the HAMD: an appetite loss group (n = 130) and a non-appetite loss group (n = 41) (**Figure 1**) (6).

#### 2.2.3 Negative life events

The Adolescent Self-rating Life Events Checklist (ASLEC) was used to assess the occurrence of negative life events in patients (18). The ASLEC includes 27 items, each of which is scored on a six-point scale. These items primarily assess six dimensions: interpersonal relationships, study pressure, punishment, sense of loss, healthy adaptation, and other factors. The higher the score on each dimension, the greater the stress associated with the relevant negative life events. Currently, the ASLEC is widely used among Chinese adolescents (19, 20).

#### 2.2.4 Alexithymia

The Toronto Alexithymia Scale (TAS-20) was used to measure individuals’ difficulties in identifying and expressing feelings (21). The scale comprises three subscales: difficulty identifying feelings, difficulty describing feelings, and externally oriented thinking. The TAS-20 consists of 20 items, each rated on a 5-point scale. The total score ranges from 20 to 100, with a higher total score indicating greater difficulties in emotional expression.

#### 2.2.5 Insomnia symptoms

The Insomnia Severity Index Scale (ISI) was used to measure the intensity of insomnia symptoms reported by participants over the previous two weeks (22, 23). This instrument comprises 7 items related to insomnia, each rated on a 5-point scale ranging from ‘0 = none’ to ‘4 = very severe’. The total ISI score ranges from 0 to 28, with higher scores indicating more severe insomnia symptoms among participants.

### 2.3 Inflammatory cytokines measurements

All subjects had their fasting blood drawn from the antecubital vein between 6:00 and 8:00 am the following day, after an overnight fast. The collected blood samples were centrifuged at 3000 rpm for 15 minutes, and the resulting plasma was extracted and stored at -80°C until analysis. The concentrations of IL-1β, IL-6, IL-10, IL-17A, and tumor necrosis factor-α (TNF-α) were measured using the Meso QuickPlex SQ120 (Meso Scale Discovery, Rockville, MD, USA). To ensure that the data for these inflammatory cytokines followed a normal distribution, we applied logarithmic transformation to the values prior to conducting statistical analyses.

### 2.4 Statistical analysis

Statistical analyses were conducted using SPSS 23.0. The Kolmogorov-Smirnov test was used to assess the normality of continuous variables. To ensure that the measurements of inflammatory cytokines conformed to a normal distribution, we referred to previous studies that performed a logarithmic transformation with a base of 10 on the levels of these cytokines to obtain Log (X) values for Log IL-1β, Log IL-6, Log IL-10, Log IL-17A and Log TNF-α (24, 25). These transformed values were used in subsequent statistical analyses. Continuous variables were summarized as mean ± standard deviation (SD) or median (quartiles) [M (P_25_, P_75_)], while categorical variables were presented as percentages (%). For univariate analyses, various statistical tests were employed, including independent samples t-tests, Mann-Whitney U-tests, and chi-square tests, to compare differences between groups with and without appetite loss across all variables. In multifactorial analyses, stepwise logistic regression was utilized to identify independent predictors of appetite loss in adolescents with MDD. And we included several confounders, including gender, age, interpersonal relationships, study pressure, sense of loss, difficulty identifying feelings and ISI score, in the analyses to control for their potential effects. All statistical tests were two-tailed, and a *p*-value of ≤ 0.05 was considered statistically significant.

## 3 Results

### 3.1 Comparison of sociodemographic and clinical characteristics of patients in appetite loss and without appetite loss groups

**Figure 2:**
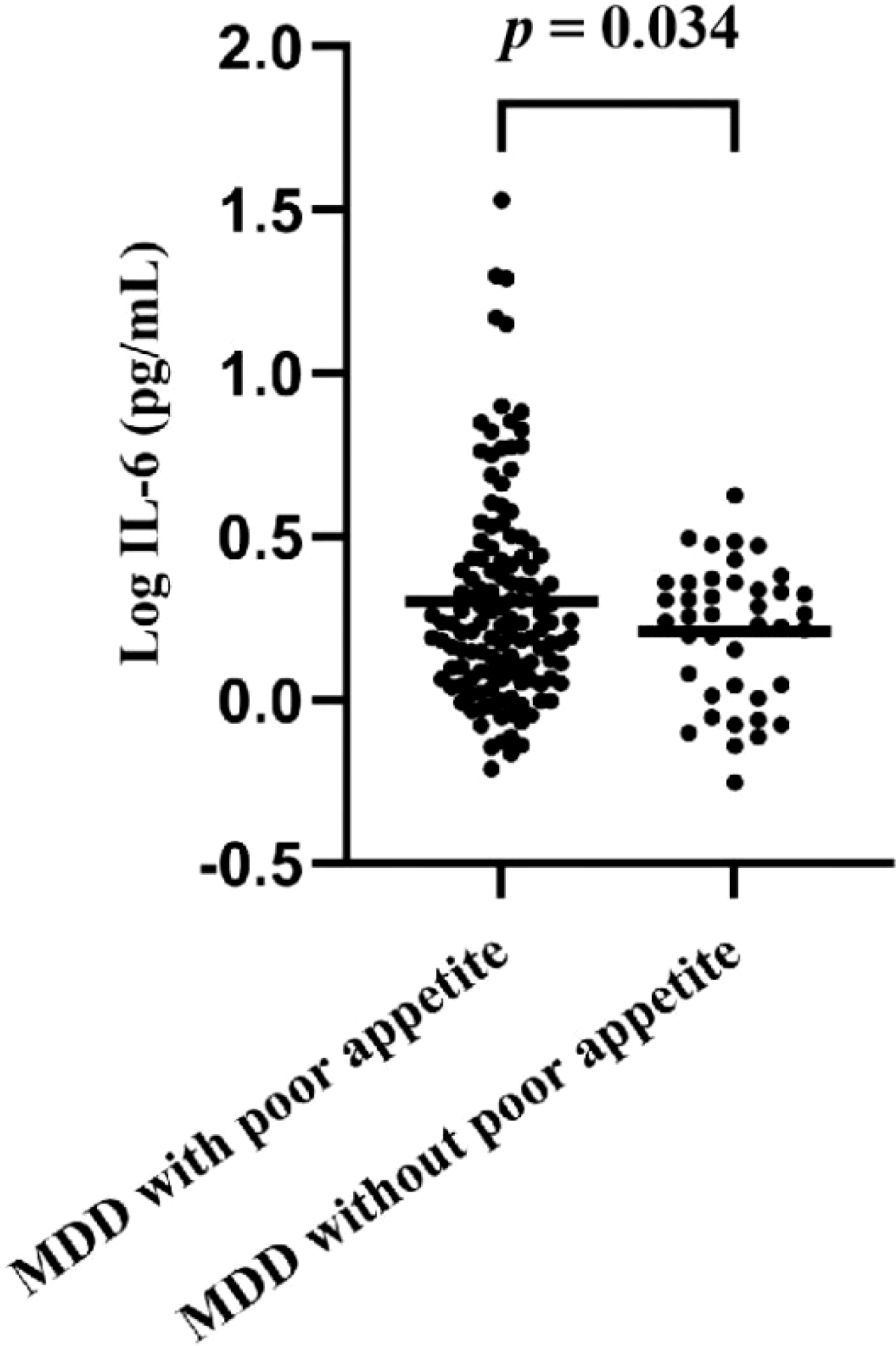
Comparison of IL-6 levels in patients with appetite loss and without appetite loss.

A total of 176 adolescents with MDD were initially recruited for this study. However, due to blood samples from an additional 5 participants not meeting the study criteria, we ended up statistically analyzing data from only 171 patients (**Figure 1**). The mean age of patients was 15.47 ± 1.44 years, and 72.5% were female. Compared to the group without appetite loss, patients with appetite loss had higher scores of HAMD (t = 6.598, *p* < 0.001), interpersonal relationships (t = 4.035, *p* < 0.001), study pressure (t = 2.167, *p* = 0.032), punishment (t = 1.858, *p* = 0.002), sense of loss (t = 1.551, *p* = 0.016), ISI (t = 3.026, *p* = 0.003) and difficulty identifying feelings (t = 3.181, *p* = 0.002), as well as higher levels of Log IL-6 (t = 2.154, *p* = 0.034) (**Table 1**; **Figure 2**).

**Table 1:** Comparison of sociodemographic and clinical characteristics of patients in appetite loss and without appetite loss groups.

Variables	Total sample (n = 171)	MDD with appetite loss (n = 130)	MDD without appetite loss (n = 41)	t/Z/χ^2^	*p*
**Females, n (%)**	124 (72.51)	99 (76.15)	25 (60.98)	3.603	0.058
**Age (years), mean (SD)**	15.47 (1.44)	15.35 (1.48)	15.85 (1.28)	-1.979	**0.049**
**BMI (kg/m^2^), mean (SD)**	21.12 (4.25)	20.87 (4.18)	21.95 (4.41)	-1.427	0.155
**Age at onset (years), mean (SD)**	13.78 (1.76)	13.68 (1.78)	14.10 (1.69)	-1.339	0.182
**Duration of illness (months), median (P_25_, P_75_)**	18.00 (12.00, 30.00)	19.00 (12.00, 31.50)	16.00 (7.50, 24.00)	0.553^a^	0.920
**Antidepressants, n (%)**				0.140	0.932
** None**	56 (32.75)	43 (33.08)	13 (31.71)		
** SSRIs**	105 (61.40)	79 (60.77)	26 (63.41)		
** Others**	10 (5.85)	8 (6.15)	2 (4.88)		
**HAMD score, mean (SD)**	28.63 (8.41)	30.76 (7.60)	21.88 (7.26)	6.598	**<0.001**
**ASLEC score, mean (SD)**					
** Interpersonal relationships**	14.45 (4.89)	15.26 (4.36)	11.88 (5.60)	4.035	**<0.001**
** Study pressure**	11.86 (4.62)	12.28 (4.45)	10.51 (4.94)	2.167	**0.032**
** Punishment**	12.00 (7.00, 16.00)	13.00 (9.00, 17.00)	7.00 (3.50, 12.00)	1.858	**0.002**
** Sense of loss**	2.00 (0.00, 5.00)	3.00 (1.00, 5.00)	1.00 (0.00, 3.00)	1.551	**0.016**
** Healthy adaptation**	6.79 (3.25)	6.94 (3.18)	6.32 (3.44)	1.069	0.286
** Other factors**	7.48 (3.73)	7.78 (3.69)	6.54 (3.75)	1.871	0.063
**TAS-20 score , mean (SD)**					
** Difficulty identifying feelings**	26.05 (5.37)	26.87 (4.75)	23.44 (6.37)	3.181	**0.002**
** Difficulty describing feelings**	18.38 (3.10)	18.64 (2.85)	17.56 (3.72)	1.955	0.052
** Externally oriented thinking**	23.50 (3.87)	23.76 (3.79)	22.66 (4.04)	1.599	0.112
**ISI score, mean (SD)**	13.20 (5.42)	13.89 (5.00)	11.02 (6.15)	3.026	**0.003**
**Log IL-1β (pg/mL), mean (SD)**	-0.54 (0.44)	-0.54 (0.45)	-0.54 (0.43)	-0.018	0.986
**Log IL-6 (pg/mL), mean (SD)**	0.28 (0.30)	0.30 (0.32)	0.21 (0.21)	2.154	**0.034**
**Log IL-10 (pg/mL), mean (SD)**	-0.24 (0.29)	-0.22 (0.30)	-0.30 (0.26)	1.409	0.161
**Log IL-17A (pg/mL), mean (SD)**	0.37 (0.33)	0.38 (0.34)	0.36 (0.29)	0.282	0.778
**Log TNF-α (pg/mL), mean (SD)**	0.08 (0.27)	0.09 (0.29)	0.06 (0.20)	0.657	0.512

MDD, major depressive disorder; BMI, body mass index; HAMD, hamilton depression scale; ASLEC, adolescent self-rating life events checklist; TAS-20, toronto alexithymia scale; ISI, insomnia severity index scale; IL, interleukin; TNF-α, tumor necrosis factor-α.

Bolded *p* value: < 0.05; SD, standard deviation.

a Mann-Whitney U test.

### 3.2 Independent factors associated with appetite loss by multivariate logistic stepwise regression analysis.

The results of multivariate logistic stepwise regression analysis were summarized in **Table 2**. The results showed that HAMD score (OR = 1.158, 95% CI = 1.091-1.229, *p* < 0.001), punishment score (OR = 1.117, 95% CI = 1.039-1.201, *p* = 0.003), and Log IL-6 level (OR = 5.041, 95% CI = 1.137-22.344, *p* = 0.033) were independent correlates of appetite loss in adolescents with MDD.

**Table 2:** Independent factors associated with appetite loss by multivariate logistic stepwise regression analysis ^a^.

Variables	B	SE	Wald χ^2^	OR	95% CI	*p*
**HAMD score**	0.147	0.030	23.463	1.158	1.091 - 1.229	**<0.001**
**Punishment**	0.111	0.037	8.983	1.117	1.039 - 1.201	**0.003**
**Log IL-6**	1.618	0.760	4.533	5.041	1.137 - 22.344	**0.033**

HAMD, hamilton depression scale; IL, interleukin.

Bolded *p* value: < 0.05; SE, standard error; OR, odds ratio; CI, confidence interval.

a The regression analyses were adjusted for several confounders, including gender, age, interpersonal relationships, study pressure, sense of loss, difficulty identifying feelings and ISI score.

## 4 Discussion

In our study, the prevalence of appetite loss among adolescents with MDD was 76.0%, significantly higher than that observed in adults with MDD (6). A case-control study showed that vegetative symptoms, including changes in appetite and weight, were more prevalent among adolescents with MDD than among adults (26). Maxwell and colleagues also identified a higher prevalence of appetite loss in adolescents with MDD through a review of empirical studies examining the associations between appetite disturbances and depressive symptoms in children, adolescents, and adults with depression (27). This phenomenon may be related to significant deficits in emotion regulation observed in adolescents with MDD (28). In addition, adolescence is a period of rapid physical and psychological changes, during which adolescents may experience dissatisfaction and anxiety regarding their body image (29). Such anxiety may stem from the internalization of societal aesthetic standards, comparisons with peers, and influences from family and media (30). When adolescents experience discomfort regarding their body size, changes in eating behaviors, such as appetite loss, may occur (31). Thus, decreased appetite in adolescents with MDD warrants high clinical priority.

In terms of clinical symptoms, this study found that patients in the appetite loss group exhibited more severe depressive symptoms. This finding was generally consistent with previous studies (11, 26). Such associations may involve complex psychological and physiological mechanisms. Firstly, regarding psychological mechanisms, depressive moods can reduce adolescents’ interest and pleasure in food, turning eating into a task rather than an enjoyment (32). For example, a survey conducted in China found that individuals with symptoms of depression and anxiety generally experienced impaired pleasure in food, particularly in its sensory experience, and may have aversions and negative expectations (33). Depressed patients often have negative self-perceptions and pessimistic attitudes towards life (34). And they may believe that they do not deserve to enjoy food or that eating does not enhance their emotional state. Regarding physiological mechanisms, depressed patients often exhibit imbalances in neurotransmitters such as serotonin and norepinephrine, which not only affect mood regulation but are also closely related to appetite regulation (35). For instance, reduced serotonin levels can result in decreased appetite (36). Additionally, orexin is an important neuropeptide involved in the regulation of physiological responses such as sleep-wakefulness and ingestion. Research has found that the orexin system becomes dysregulated in MDD, thereby affecting appetite regulation (37). Finally, disturbances of the endocrine system in depressive states, such as abnormally elevated cortisol levels, can also affect appetite and energy metabolism, leading to appetite loss (38).

Also, we found that a series of negative life events (especially punishment) were significantly associated with appetite loss in adolescents with MDD. Previous studies have well established that various types of negative life events are significantly associated with depression in adolescents (39). Given the strong association between depressive symptoms and appetite loss, negative life events may lead to appetite loss in adolescents through their impact on depressive symptoms. Psychological stress resulting from negative life events may also influence appetite in adolescents with MDD (40). Moreover, negative life events can trigger intense negative emotions such as anxiety, fear and despair (41). These emotions may disrupt normal appetite regulation mechanisms, causing individuals to prioritize coping with emotional stress over satisfying physiological needs (42). For example, the experience of punishment can induce feelings of shame and self-blame in adolescents. This psychological burden can cause discomfort during meals, leading to reduced food intake. Consequently, clinicians should closely monitor the dietary status of adolescents with MDD who have experienced adverse life events.

As well, insomnia and alexithymia might be associated with appetite loss in adolescents with MDD, although regression analyses did not show statistical significance. We speculated that insomnia might lead to physical and psychological fatigue, which could disrupt appetite regulation mechanisms and decrease interest in food. Additionally, insomnia may also lead to an increased stress response in the body, disrupting the normal functioning of the hypothalamic-pituitary-adrenal (HPA) axis and causing appetite loss (43). Most research on the association between alexithymia and appetite has focused on patients with eating disorders. A meta-analysis of 44 studies showed that patients with all types of eating disorders exhibit more pronounced symptoms of alexithymia, particularly in terms of difficulty identifying or describing feelings (44). We speculated that patients with alexithymia might be prone to prolonged negative emotional states due to difficulties in recognizing and expressing emotions, and that these negative emotions might suppress appetite. Of course, more research is needed to confirm this hypothesis.

Of note, patients in the appetite loss group exhibited higher levels of plasma inflammatory cytokines (particularly IL-6) in this study. Previous studies conducted with different populations have yielded similar findings (6, 15, 45, 46). A study of patients with end-stage cancer found that plasma IL-6 levels were positively correlated with appetite loss (45). Another network analysis study also showed that various depressive symptoms, including feelings of worthlessness and poor appetite, were associated with IL-6 in adolescents (46). The factors mediating the associations between inflammatory cytokines and appetite loss are likely multifaceted. In fact, some cytokines (e.g. IL-6 and TNF-α) have anorexic properties themselves and can directly influence appetite regulatory centres (47, 48). Research has also shown that inflammatory cytokines can stimulate the release of leptin, an anorexigenic peptide that suppresses appetite (49, 50). In addition, pro-inflammatory cytokines can suppress appetite by activating the HPA axis and the sympathetic nervous system, resulting in elevated levels of cortisol and norepinephrine (49, 51). Finally, pro-inflammatory cytokines can induce anhedonia, a core symptom of depression directly related to appetite loss (52). Notably, in the present study, IL-6 showed a more significant correlation with appetite loss in adolescents with MDD. This phenomenon may stem from the unique role of IL-6 in appetite regulation. Compared with other cytokines such as TNF-α and IL-1, the role of IL-6 in appetite regulation may be more critical. Firstly, IL-6 is able to pass through the blood-brain barrier (BBB) or act in areas where the BBB is weak (e.g., the posterior hypothalamic region), directly or indirectly affecting the appetite-regulating centers in the hypothalamus (53). Specifically, IL-6 can activate neuropeptides such as corticotropin-releasing hormone (CRF) in the hypothalamus and inhibit the activity of the appetite-stimulating neuropeptide Y (NPY), which leads to a decrease in appetite (54). In addition, although a variety of cytokines are involved in the inflammatory response, IL-6 may play a more critical role in certain specific inflammatory states (e.g., chronic low-grade inflammation) (55). This inflammatory state may be more closely associated with appetite loss in adolescents with MDD. However, relatively few studies have been conducted on the relationship between appetite loss and IL-6 in adolescents with MDD, so it is necessary to further explore this topic in the future to clarify the specific mechanism of IL-6’s role in appetite loss in this special population and to provide a theoretical basis for clinical intervention.

However, there were some limitations in this study: (1) The sample was drawn from only two healthcare organizations, and the sample size was relatively small, which might limit the generalizability of the results. (2) This study utilized a cross-sectional design, which did not support explicit inferences about causality. Therefore, future longitudinal or interventional studies are necessary to further elucidate the causal relationship between appetite loss and inflammatory cytokines in adolescents with MDD. (3) The assessment of appetite loss in this study was based solely on one item of the HAMD scale, which may introduce bias. Therefore, future validation of these findings using a more reliable tool is necessary. (4) Previous studies have shown that antidepressant medications and certain personality traits, such as impulsivity, can also affect appetite (56, 57). Therefore, future research should include additional variables to fully explore the factors influencing appetite loss in adolescents with MDD.

## 5 Conclusion

There was an elevated risk of appetite loss among adolescents with MDD. The present study suggested that appetite loss might be associated with a range of clinical symptoms (especially depression severity and negative life events) and levels of inflammatory cytokines (especially IL-6). Therefore, to effectively prevent and ameliorate appetite loss in adolescents with MDD, healthcare professionals should actively intervene to address relevant risk factors, such as modulating inflammation levels. Additionally, future research should include more high-quality prospective studies to provide in-depth mechanistic insights into appetite loss in this population.

## Data Availability

The raw data supporting the conclusions of this article will be made available by the authors, without undue reservation.
